# Design and rationale of ischaemia-driven complete revascularisation versus usual care in patients with non-ST-elevation myocardial infarction and multivessel coronary disease: the South Limburg Myocardial Infarction (SLIM) trial

**DOI:** 10.1007/s12471-019-01332-w

**Published:** 2019-09-17

**Authors:** T. F. S. Pustjens, B. Streukens, J. Vainer, B. Gho, A. W. Ruiters, M. Stein, M. Ilhan, L. Veenstra, R. Theunissen, S. C. A. M. Bekkers, A. W. J. van’t Hof, S. Rasoul

**Affiliations:** 1 Department of Cardiology, Zuyderland Medical Centre, Heerlen, The Netherlands; 2 Department of Cardiology, Maastricht University Medical Centre, Maastricht, The Netherlands; 3 Department of Cardiology and Radiology, Maastricht University Medical Centre, Maastricht, The Netherlands

**Keywords:** Acute coronary syndrome, Non-ST-elevation myocardial infarction, Multivessel disease, Complete revascularisation

## Abstract

**Aims:**

To compare ischaemia-driven complete coronary revascularisation by percutaneous coronary intervention (PCI) with usual care in patients with non-ST-elevation myocardial infarction (non-STEMI) and multivessel disease (MVD).

**Methods:**

The South Limburg Myocardial Infarction (SLIM) trial (NCT03562572) is an investigator-initiated, prospective, multicentre, randomised controlled trial that compares fractional flow reserve (FFR)-guided complete revascularisation during the index procedure with usual care in non-STEMI patients with MVD. A total of 414 patients will be randomised in a 1:1 fashion. The primary endpoint is the composite of all-cause mortality, non-fatal myocardial infarction, and any revascularisation and stroke (MACCE) at 12 months. The secondary endpoints are: MACCE at 24 and 36 months, and the composite of cardiac death, myocardial infarction, any revascularisation, stroke, major bleeding and left ventricular ejection fraction below 45% at 12, 24 and 36 months. Furthermore, quality of life will be assessed by the Patient Health Questionnaire (PHQ-9) and the Short Form (36) Health Survey (SF-36) at 1 and 12 months of follow-up.

**Conclusion:**

The SLIM trial aims to provide evidence whether FFR-guided complete revascularisation by PCI is superior to usual care with respect to clinical outcomes (major adverse cardiovascular events) in non-STEMI patients with MVD.

## Introduction

Patients with ST-elevation myocardial infarction (STEMI) and multivessel disease (MVD) have a worse prognosis than STEMI patients with single-vessel disease [1]. Several randomised clinical trials and meta-analyses have shown that, compared with infarct-related artery only PCI (IRA-PCI), multivessel percutaneous coronary intervention (MV-PCI) resulted in fewer major adverse cardiovascular events (MACE), mainly driven by a reduction in repeat revascularisation [2–5].

Compared with STEMI patients, patients with non-ST-elevation myocardial infarction (non-STEMI) have a higher risk profile, a higher incidence of MVD and less favourable outcome [6, 7]. However, there is no clear evidence regarding the role of ischaemia-driven complete coronary revascularisation by PCI in patients with non-STEMI during the index procedure. Furthermore, both the American and the European guidelines are unclear as to which coronary revascularisation strategy is preferred in non-STEMI patients with MVD [8, 9].

Previous studies showed that MV-PCI in non-STEMI patients reduces follow-up revascularisation rates without affecting MACE [10–12]. However, others also found MACE reduction after MV-PCI [13, 14]. In the SMILE (Survival of Myocardial Infarction Long-term Evaluation) trial, MV-PCI during the index procedure in non-STEMI patients with MVD was superior to multistage PCI during the index hospitalisation in terms of major adverse cardiovascular and cerebrovascular events (MACCE). This was mainly due to repeat coronary revascularisation, without a significant effect on cardiac death and reinfarction [14]. In the above-mentioned trials, assessment of lesion severity was mostly performed visually and fractional flow reserve (FFR) measurements were only performed in approximately 25% of the patients.

Currently, FFR-guided revascularisation has become the standard for in-catheterisation laboratory assessment of flow-limiting lesions in patients with stable angina, but has also been shown to be reliable in patients with unstable angina and non-STEMI [15–17]. The beneficial effect of FFR-guided MV-PCI compared with standard angiography was proven in the large randomised, multicentre FAME (Fractional Flow Reserve Versus Angiography for Multivessel Evaluation) study [18, 19]. In that study, it was demonstrated that all types of adverse events were decreased by 30% in the 1st year after PCI when guided by FFR. The information provided by FFR is similar to that obtained with myocardial perfusion studies. However, it is more specific and masking of one ischaemic area by another, more severely ischaemic, zone is avoided since every artery or segment is analysed separately [20, 21]. In addition, FFR-guided revascularisation has been shown to be cost-effective [22]. A recent study has shown that FFR can reliably assess the haemodynamic severity of non-culprit coronary artery stenosis during the acute phase of STEMI [23]. Therefore, FFR may offer the same advantages in the decision-making process on revascularisation of the non-culprit artery in patients presenting with non-STEMI, similarly as in stable coronary syndromes.

Taking the above into consideration, we expect that ischaemia-driven (FFR) complete percutaneous revascularisation of all significant stenoses in lesions of non-culprit arteries performed within the index PCI procedure will improve clinical outcomes compared to the usual care, guided by the discretion of the physician.

## Methods

### Study design

The South Limburg Myocardial Infarction (SLIM) trial is an investigator-initiated, multicentre, prospective, randomised clinical trial in non-STEMI patients with multivessel coronary artery disease amenable to treatment with PCI, in which 414 consecutive patients will be randomised in a 1:1 fashion, after completion of a successful culprit lesion PCI. In the IRA-PCI the identification of the culprit vessel will be performed according to electrocardiographic, echocardiographic and angiographic parameters as stated in the ESC guidelines [24]. The use of anatomic (intravascular ultrasound or optical coherence tomography) or functional (FFR) imaging modalities to assess the culprit lesion, or to rule out other mechanisms such as dissection or haematomas, will be left to the operator’s discretion. A patient will be excluded if the culprit artery is uncertain. Radial access will be strongly recommended for performance of coronary angiography and PCI.

**Fig. 1:**
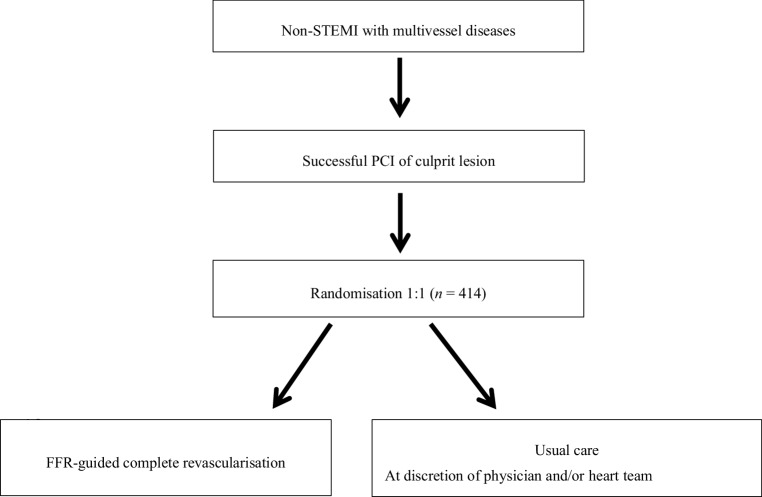
Randomisation procedure. *FFR* fractional flow reserve, *Non-IRA* non-infarct-related artery, *Non-STEMI* non-ST-elevation myocardial infarction, *PCI* percutaneous coronary intervention

All patients from June 2018 who present with at least one lesion with a stenosis of approximately 50% or more in a non-IRA with a diameter of ≥2.0 mm and fulfil the inclusion and exclusion criteria will be enrolled. Inclusion and exclusion criteria are listed in Tab. 1. The estimated duration of enrolment is 3 years. An overview of the trial is presented in Fig. 1.

**Table 1:** Inclusion and exclusion criteria

Inclusion criteria	Exclusion criteria
Age between 18 and 85 years	Left main coronary artery disease (stenosis >50%)
Presenting with non-STEMI, to be treated with PCI of culprit lesion	Chronic total occlusion of a non-IRA
Presence of at least one stenosis of >50% in a non-IRA on QCA or visual estimation of baseline angiography	Complicated IRA treatment, e.g. extravasation, permanent no reflow after IRA treatment (TIMI flow 0–1) and inability to implant a stent
Non-IRA stenosis amenable for PCI treatment (operator’s decision)	Indication for or previous coronary artery bypass grafting
Able and willing to give signed informed consent	Known severe cardiac valve dysfunction requiring surgery or TAVI in follow-up period
	Killip class III or IV during completion of culprit lesion treatment
	Uncertain culprit lesion
	Life expectancy of <1 year
	Intolerance to aspirin, clopidogrel, prasugrel, ticagrelor or heparin
	Gastrointestinal or genitourinary bleeding in previous 3 months
	Planned elective surgical procedure necessitating interruption of P2Y12 inhibitors during first 6 months post-enrolment
	Currently enrolled in another clinical trial
	Pregnancy
	Expected loss to follow-up

*IRA* infarct-related artery, *PCI* percutaneous coronary intervention, *STEMI* ST-elevation myocardial infarction, *TAVI* transcatheter aortic valve implantation, *TIMI* thrombolysis in myocardial infarction, *QCA* quantitative coronary angiography

The study has been approved by the Medical Ethical Committee of Zuyderland Medical Centre, The Netherlands. The SLIM trial is registered at ClinicalTrials.gov (NCT03562572) and the Netherlands Trial Register (NTR7158).

### Informed consent and randomisation

Informed consent will be obtained according to the Good Clinical Practice guidelines. To participate in the trial, the participant must give oral approval before randomisation in the presence of a third independent (not involved in the study) person (catheterisation laboratory personnel or a nurse) after successful PCI of the culprit. To avoid a further delay in treatment, a signature will be obtained after full completion of the procedure irrespective of the treatment allocation, but must be obtained before the patient leaves the catheterisation laboratory. If angiographic inclusion criteria are met (presence of at least one stenosis of >50% in a non-IRA with a diameter of ≥2.0 mm) and PCI of the IRA is successful, the patient will be randomised via an online randomisation tool. If the patient is randomised to the usual care group, he or she will be informed that all intermediate lesions will be discussed in the heart team as to whether or not additional revascularisation of the non-IRA arteries will be performed. All patients will receive medical treatment based on current guidelines.

### Ischaemia-driven complete revascularisation group

In the ischaemia-driven complete revascularisation strategy group all flow-limiting (FFR ≤0.80) lesions will receive treatment by PCI and stenting. The non-IRA PCI should be performed during the same intervention. Exceptions can be made for complex lesions where the operator estimates that the revascularisation procedure will require significant contrast overload, which may lead to deterioration of cardiac and renal function of the patient. In such cases, a second procedure should be performed, which should take place within the same hospitalisation, preferably within 72 h.

### Usual care group

In the randomised to usual care group the procedure will stop after the PCI of the culprit artery and the patient will be referred to his/her treating cardiologist and/or heart team. They will decide whether or not (ischaemia-driven) staged PCI of the non-IRA arteries should take place. The following treatment options are possible: (1) FFR- or iFR- (instantaneous wave-free ratio) guided PCI of the non-IRA arteries, (2) additional non-invasive tests, (3) symptom-driven PCI of the non-IRA arteries, or (4) optimal medical therapy. If the treating cardiologist (after consulting the heart team) decides to perform the non-IRA PCI revascularisation, then such treatment should take place within 6 weeks of the primary PCI in order to count as a scheduled staged PCI procedure. Any other revascularisations of any lesions after these 6 weeks are identified as unscheduled and therefore counted as an event.

All revascularisation procedures will be defined as clinically indicated or not clinically indicated by the independent clinical events committee.

## Study endpoints

### Primary endpoint

Primary study endpoints are defined as the incidence of MACE (composite endpoint of all cause death, non-fatal myocardial infarction, any revascularisation and stroke) at 12 months.

### Secondary endpoints

- Primary endpoint in subgroups at 12 and 24 months.
- Composite endpoint of net adverse clinical events defined as composite endpoint of cardiac death, myocardial infarction, any revascularisation, stroke and major bleeding at 12, 24 and 36 months.
- Composite endpoint hospitalisation for heart failure and unstable angina pectoris at 12, 24 and 36 months.
- All-cause mortality or myocardial infarction at 12, 24 and 36 months.
- Any revascularisation at 12, 24 and 36 months.
- Stent thrombosis at 12, 24 and 36 months.
- Bleeding (major and minor) at 48 h and 12 months.
- Primary endpoint at 36 months as well as outcomes of each component of the primary endpoint at 12 and 24 and 36 months.
- Left ventricular ejection fraction below 45% at 12, 24 and 36 months (myocardial perfusion scintigraphy, magnetic resonance imaging or echocardiography).
- Quality of life at 1 and 12 months measured by the Patient Health Questionnaire (PHQ-9) and the Short Form (36) Health Survey (SF-36)

## Follow-up

For endpoint adjudication office-based direct visits will be performed at 1 and 12 months, and telephone-based interviews will be performed at 24 and 36 months. At 1 and 12 months of follow-up, quality of life will be evaluated by the PHQ‑9 and the SF-36.

## Statistical analysis

### Sample size calculation and statistical power analysis

The event rates for both groups were defined as follows:

In the usual care group, the event rates are estimated based on data from our previous all-comers registry [7] and the SMILE trial [13]. Rates of MACE in the ischaemia driven group are estimated based on the results of the SMILE trial [13].

In order to assess the superiority of MV-PCI over IRA-PCI, we assume the incidence of MACE to be 20% in the IRA-PCI group and 10% in the ischaemia-driven MV-PCI group. On the basis of a two-sided test size of 5% and a power of 80%, it is calculated that a minimum of 197 patients is needed to be recruited in each group to detect a 10% difference in the incidence of MACE at 1 year. To account for 5% loss to follow-up, a total of 414 (394/0.95) patients will be recruited.

The primary analysis is an intention-to-treat analysis of all randomised patients. A per-protocol analysis will also be performed. All continuous variables will be expressed as mean ± SD and analysed by the Student’s *t*-test. Categorical variables will be analysed by the chi-square or Fisher exact test, as appropriate. Baseline variables with non-Gaussian distributions will be compared using the Mann-Whitney U test and summarised with medians and interquartile range. For the primary endpoint and its components, 95% confidence intervals will be reported. The event-free survival curve for MACE will be constructed using the Kaplan-Meier method, and statistical differences between curves will be assessed by the log-rank test. The hazard ratio for treatment comparisons will be estimated using Cox proportional hazard models. Mean scores of the health-related quality of life will be compared between groups using the independent *t*-test. Statistical analysis will be performed with SPSS (version 15.0, SPSS, Chicago, IL, USA). An independent endpoints committee blinded to the randomisation group will adjudicate clinical study endpoints.

Descriptive analyses of primary outcomes will be performed for the following pre-specified subgroups:

1. Diabetic patients versus non-diabetic patients
2. Elderly (≥75 years) versus young patients (<75 years)
3. Male versus female gender
4. High- (GRACE >140) versus low-risk (GRACE ≤140) patients according to Global Registry of Acute Coronary Events (GRACE) risk score
5. Patients with previous myocardial infarction versus patients with no previous myocardial infarction

### Interim analysis

An interim analysis will be performed 6 months after 150 patients have been included. The interim analysis will be performed on the safety population and the actual treatment group will be unblinded to the independent statistician. All patients who are accrued and treated will be included in the analysis. Events of patients who are lost to follow-up, or who do not have 6 months of follow-up, will be included. The difference between groups in proportion of patients with events at 6 months of follow-up will be tested by means of a one-sided Fisher exact test for differences in independent binomial proportions. The data safety monitoring board will use the following stopping rule: If there is a significant difference in MACE between the two groups the trial will be stopped (one-sided test, alpha = 0.05).

## Trial coordination

An independent Clinical Trial Centre will monitor the SLIM trial. The study monitor will visit each site at appropriate intervals to review investigational data for accuracy and completeness and ensure compliance with the protocol.

The study will be overseen by a trial steering committee and an independent Data and Safety Monitoring Board will review the clinical outcome data.

## Conclusion

The SLIM trial is a prospective, multicentre, 1:1 randomised controlled trial in which 414 patients with non-STEMI and MVD will be included. The study aims to provide evidence whether FFR-guided complete revascularisation by PCI is superior with respect to clinical outcomes compared to usual care in non-STEMI patients with MVD.
